# Infection with *Plasmodium berghei* ookinetes alters protein expression in the brain of *Anopheles albimanus* mosquitoes

**DOI:** 10.1186/s13071-016-1830-9

**Published:** 2016-10-11

**Authors:** Alejandro Alvarado-Delgado, Guillermo Perales Ortiz, Ángel T. Tello-López, Sergio Encarnación, Renaud Conde, Ángel G. Martínez-Batallar, Ken Moran-Francia, Humberto Lanz-Mendoza

**Affiliations:** 1 Centro de Investigación Sobre Enfermedades Infecciosas, Instituto Nacional de Salud Pública, Av. Universidad 655, C. P. 62100 Cuernavaca, Morelos México; 2 Centro de Ciencias Genómicas, Universidad Nacional Autónoma de México, Cuernavaca, México

**Keywords:** *Plasmodium*, Ookinete, *Anopheles albimanus*, Neuron, Proteomic, Brain

## Abstract

**Background:**

The behaviour of *Anopheles* spp. mosquitoes, vectors for *Plasmodium* parasites, plays a crucial role in the propagation of malaria to humans. Consequently, it is important to understand how the behaviour of these mosquitoes is influenced by the interaction between the brain and immunological status. The nervous system is intimately linked to the immune and endocrine systems. There is evidence that the malaria parasite alters the function of these systems upon infecting the mosquito. Although there is a complex molecular interplay between the *Plasmodium* parasite and *Anopheles* mosquito, little is known about the neuronal alteration triggered by the parasite invasion. The aim of this study was to analyse the modification of the proteomic profile in the *An. albimanus* brain during the early phase of the *Plasmodium berghei* invasion.

**Results:**

At 24 hours of the *P. berghei* invasion, the mosquito brain showed an increase in the concentration of proteins involved in the cellular metabolic pathway, such as ATP synthase complex alpha and beta, malate dehydrogenase, alanine transaminase, enolase and vacuolar ATP synthase. There was also a rise in the levels of proteins with neuronal function, such as calreticulin, mitofilin and creatine kinase. Concomitantly, the parasite invasion repressed the expression of synapse-associated proteins, including enolyl CoA hydratase, HSP70 and ribosomal S60 proteins.

**Conclusions:**

Identification of upregulated and downregulated protein expression in the mosquito brain 24 hours after *Plasmodium* invaded the insect midgut paves the way to better understanding the regulation of the neuro-endocrine-immune system in an insect model during parasite infection.

**Electronic supplementary material:**

The online version of this article (doi:10.1186/s13071-016-1830-9) contains supplementary material, which is available to authorized users.

## Background

Anopheline mosquitoes represent the principal vectors for transmission of protozoan parasites of the genus *Plasmodium* to humans. In 2015, 215 million malaria cases were reported worldwide, causing 438,000 deaths [1]. To control this public health problem, it is necessary to deepen the understanding of the interaction of this insect species with the parasite.

The molecular interplay between *Anopheles* and *Plasmodium* has been extensively studied in regard to the immune response of the mosquito when invaded by the parasite [2–4] and the molecular mechanisms of evasion developed by the latter [5]. Previous reports have shown that *Plasmodium falciparum* and *P. yoelii nigeriensis* alter the behaviour of their hosts, *Anopheles gambiae* and *An. stephensi*, respectively [6, 7]. Specifically, infection of mosquitoes causes a greater insect contact rate with the vertebrate host [8, 9] because of the increase in the number of blood-feedings [9]. At the same time, enzymatic activity in the mosquito salivary glands diminishes [10].

Since the mosquito brain and nervous system dictate feeding behaviour, muscle activity, neuropeptide and hormonal secretion, as well as others functions [11], they are expected to play a crucial role in malaria dissemination. Moreover, there is cross-talk between the immune and nervous systems, which occurs through a complex set of neurotransmitters, cytokines, hormones and neuropeptides [12]. The neural response to a parasite invasion could be generic and stress dependent or pathogen-specific. The pathway of infection-related signal transmission from midgut cells to the brain is still unknown. The effects of midgut cell invasion by *Plasmodium* ookinetes, and the consecutive shedding reaction may generate stress signals that are perceived by mosquito brain cells. Signals related to energy deprivation as well as oxidative and immune stress are transmitted throughout the organism. Our group has demonstrated that when midguts of *An. albimanus* are invaded by *P. berghei* ookinetes, they produce and release nitric oxide (NO) and hydrogen peroxide (H_2_O_2_) at 24 h post-infection, compounds that are able to activate a systemic immune response [13]. Furthermore, the presence of the parasite in the *An. albimanus* midgut induces changes in the protein profile of midgut cells [14].

In the early stage of the ookinete invasion of mosquito midgut tissues (24 h post-intake of infected blood meal), there is an alteration in the expression of immunity/defence, mitochondrial redox, metabolism, and transcription/regulation of proteins in these tissues [14]. However, the changes occurring in the mosquito neural system during the early phases of *Plasmodium* infection are as yet scarcely explored. Considering that the mosquito brain regulates feeding conduct as well as muscular activity, the analysis of its proteome may help unveil the mechanisms underlying the behavioural changes of mosquitoes that have been observed during a *Plasmodium* infection. A differential head proteome of sporozoite-infected versus uninfected anopheline mosquitoes revealed that various proteins associated with metabolism, synaptic transmission, heat-shock response, signalling, and cytoskeleton function were altered by the presence of the parasite 20 days post-infection [15]. We herein used a proteomic approach to analyse protein expression in the mosquito brain 24 h after feeding on blood infected with *P. berghei* ookinetes. Compared to the brain of control mosquitoes, we observed modifications in the protein expression of different physiological processes at 24 h post-invasion of midgut cells by ookinetes. The current results shed light on the early processes taking place in the mosquito brain during an ookinete invasion of the midgut.

## Methods

### Mosquitoes and infection with *P. berghei*

White stripe strain *An. albimanus* females [16] were obtained from the insectary of the National Institute of Public Health (INSP) in Cuernavaca, Mexico. Mosquitoes were bred under a 12:12 photoperiod at 28 °C and 70–80 % relative humidity. To determine that the changes in the proteins expression were mainly due to the presence of *Plasmodium* in the midgut, mosquitoes were treated with antibiotics [17]. They had *ad libitum* access to food, which was 8 % sucrose with PSN 1× (5,000 U of penicillin, Streptomycin at 5 mg/ml, and Neomycin at 10 mg/ml) and gentamicin (50 μg/ml) (Thermo Fisher Scientific, Waltham, Massachusetts, USA) absorbed in cotton pads and provided during the 72 h before infection. Cotton pads were changed daily. This antibiotic treatment almost eliminates all bacteria in the midgut of *An. albimanus* [17]. At 4 days post-emergence, mosquitoes were infected with *P. berghei* ANKA expressing the green fluorescent protein (GFP) [18] (kindly donated by Robert E. Sinden, Imperial College, UK).

Ookinetes were produced by culturing gametocyte-infected mouse blood, as described previously [19]. Groups of 300 female mosquitoes were fed for 1 h using artificial membrane feeders with: (i) mouse blood + GFP ookinetes (infected group, with approximately 800 ookinetes per μl), or (ii) mouse blood only (control group). Unfed mosquitoes were removed and the engorged ones incubated at 21 °C to allow for parasite invasion and interaction with the mosquito midgut. Ookinetes recognize, interact with and invade the midgut cells.

### Brain samples

Brains were extracted from mosquitoes at 24 h post-feeding. Mosquitoes were cold anesthetized for 10 min at 4 °C and maintained on ice. Three hundred brains were obtained from each group of mosquitoes. Tissues were kept in 200 μl PBS 1× with protease inhibitors (2 mM PMSF, 2 mM TLCK, 0.1 mg/ml leupeptin and 1 mM EDTA) (Sigma-Aldrich, St. Louis, Missouri, USA) and stored at -70 °C to await further use. Midguts were analysed under a fluorescence microscope to confirm the presence (infected group) or absence (control group) of ookinetes. In the former case, only tissues from mosquitoes infected with *P. berghei* were used for the analysis of expression. Prior to first dimension IEF, the brains were solubilised in a solution containing 7 M urea, 100 mM DTT, 2 % CHAPS and 0.2 % ampholine (ampholine lysis buffer; Sigma-Aldrich). The protein content was determined using the BCA method (Thermo Fisher Scientific). Triplicates of 125 μg of control and experimental samples were separated on individual IEF strips.

### 2D electrophoresis and image analysis

First dimension IEF was carried out in an Ettan IPGphor 3 unit as described by the manufacturer (Amersham Biosciences, Uppsala, Sweden). For the isoelectric focusing separation, 13 cm, pH 3–10 NL (nonlinear) precast IPG strips were submitted to 2000 V tension for 17 h at 20 °C, as per the manufacturer’s instructions. Prior to SDS-page second dimension separation (2-D), the IPG strips were placed in the rehydration tray and the proteins in the strip were reduced and alkylated by sequential incubation in the following solutions: 0.04 M Tris-HCl, pH 6.8, 1 % SDS; 30 % glycerol (equilibration buffer-EB) supplemented with 4 mg/ml DTT in EB; and then 40 mg/Ml iodoacetamide in EB. After isoelectric focusing, the IPG strips were applied directly to 13 % SDS-polyacrylamide gels for second-dimension electrophoresis at 150 V in an Owl electrophoretic chamber (Thermo Fisher Scientific). The gels were fixed and then stained with PhastGel Blue (Amersham Biosciences), according to the manufacturer’s instructions. The stained gels were digitalized using a GS-800 densitometer (Bio-Rad Hercules, CA, USA). Images from 2-D gels of three biologically independent protein extracts from control and experimental group were processed using the PD-Quest 8.0.1 software (Bio-Rad). Protein spots in all replicates were detected automatically by the software, and the detection was improved by the manual addition of omitted spots and the elimination of incorrectly detected spots. Normalization of gel images was performed using the local regression model normalization method supply by PD-Quest software. To appropriately compare the samples, the gel images were adjusted to fit a common distortion model; this was done by matching spots that were common to all the gel images. The gel images from the different protein samples were compared to each other in order to generate a robust data set containing all the spots represented in the samples with 98 % statistical confidence (*P* < 0.01) in a Student׳s t-test. Lastly, the protein spots in the statistical data set displaying ± 2-fold abundance change were selected and then submitted to mass spectrometry (MS) identification.

### Protein proteolysis, MS identification and functional annotation

Selected spots from Coomassie blue-stained two-dimensional gels were excised manually and frozen at -70 °C until further use. Samples were prepared for mass spectrum analysis using a slight modification of a previously described procedure [20, 21]. Protein spots were destained, reduced, alkylated and digested with trypsin (Promega, Madison, WI, USA). Before the mass spectra of the peptide mixtures were obtained, these solutions were desalted using a C18 Zip Tip (Millipore, Bedford, MA, USA), according to the manufacturer’s recommendations.

Mass spectra were determined using a Bruker Daltonics Autoflex (Bruker Daltonics, Billerica, MA, USA) operated in the delayed extraction and reflectron mode. Spectra were externally calibrated using a peptide calibration standard (Bruker Daltonics). Peptide mixtures were analysed using a saturated solution of alpha-cyano-4-hydroxycinnamic acid in 50 % acetonitrile-0.1 % trifluoroacetic acid. Lists of the peaks of tryptic peptide masses were generated and searched against the NCBI nr (NCBI, Maryland, USA; https://www.ncbi.nlm.nih.gov/refseq/about/nonredundantproteins/#reference) [22] and a anopheline protein data bank (https://www.vectorbase.org/organisms) [23] using the Mascot search program (Matrix Science, London, UK; http://www.matrixscience.com) [24], with the following parameters: one missed cleavage allowed, carbamidomethylcysteine as the fixed modification, and oxidation of methionine as the variable modification. We accepted proteins with scores > 50 and *P* < 0.05.

Protein interaction networks, built using the online database resource Search Tool for the Retrieval of Interacting Genes (STRING) [25], were visualized by Medusa [26], a Java application for visualizing and manipulating graphs of interaction. The interactions include direct (physical) and indirect (functional) associations derived from the genomic context, high-throughput experiments, co-expression and literature mining.

## Results

### Two-dimensional (2D) gel electrophoresis pattern analysis

**Fig. 1:**
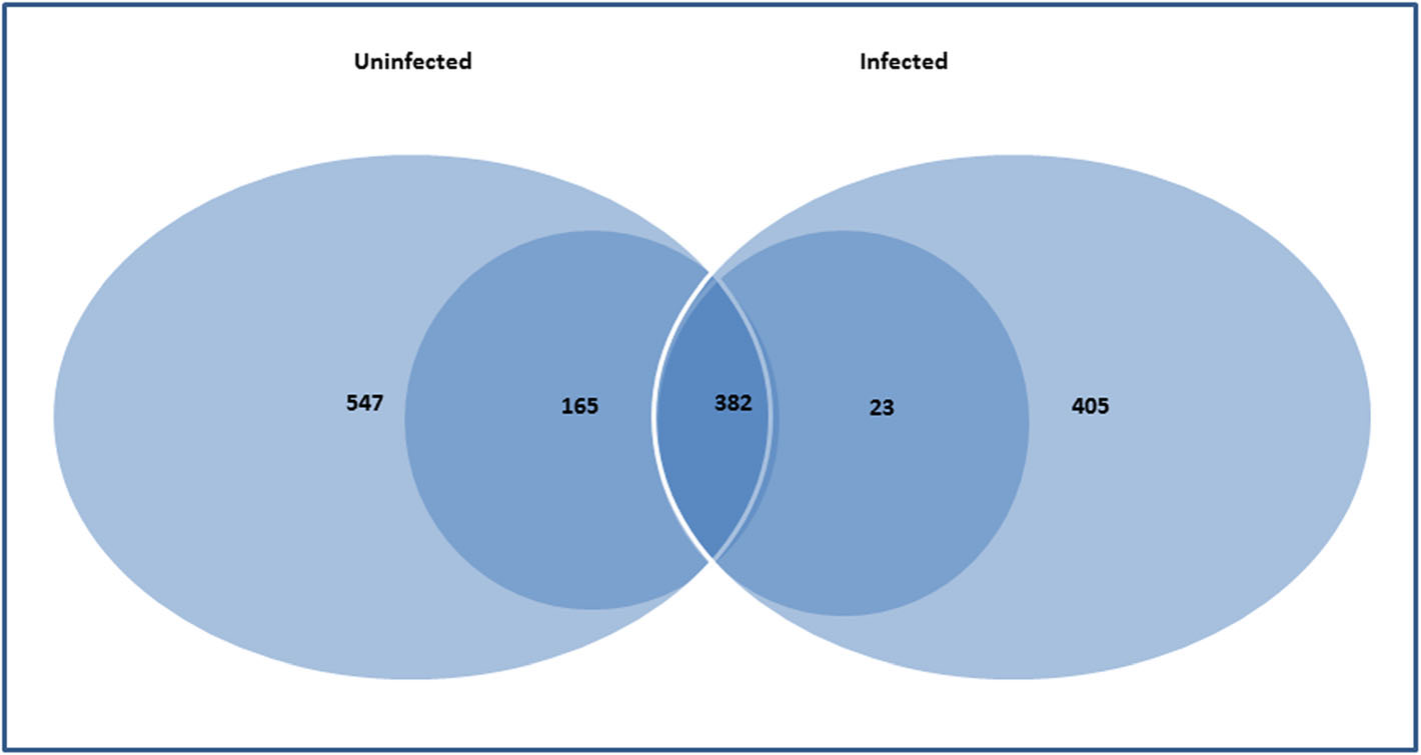
Venn diagram depicting total spots detected in control (547) and infected brains (405) in *An. albimanus*: 382 spots were present in both conditions, while 165 were found only in the control group and 23 only in the infected group

Brain tissues of the infected and control group of mosquitoes were examined to determine whether the ookinete infection changes protein expression patterns. 2D-gel electrophoresis of both groups showed an extensive protein pattern. Five hundred and forty-seven and 405 well-resolved Coomassie stained spots were detected in the control and infected group, respectively. Three hundred and eighty-two spots were present in both groups (Fig. 1). The molecular weight of these spots varied from 15 to 130 kDa, with a range in the isoelectric point (pI) of 2.0 to 8.9.

### Differential brain protein expression between control and infected mosquitoes

The average protein levels in the spots were quantified, and those showing relative changes in abundance of > 2-fold between conditions (increase or decrease) at the 99 % confidence level (*P* < 0.01) were considered significantly different (for details see Methods). A total of the 188 differential spots were observed there were 165 in the control (88 %) and 23 (12 %) in the infected group, evidencing that the *Plasmodium* midgut tissue invasion does trigger a brain response (Fig. 1).

### Identification

**Fig. 2:**
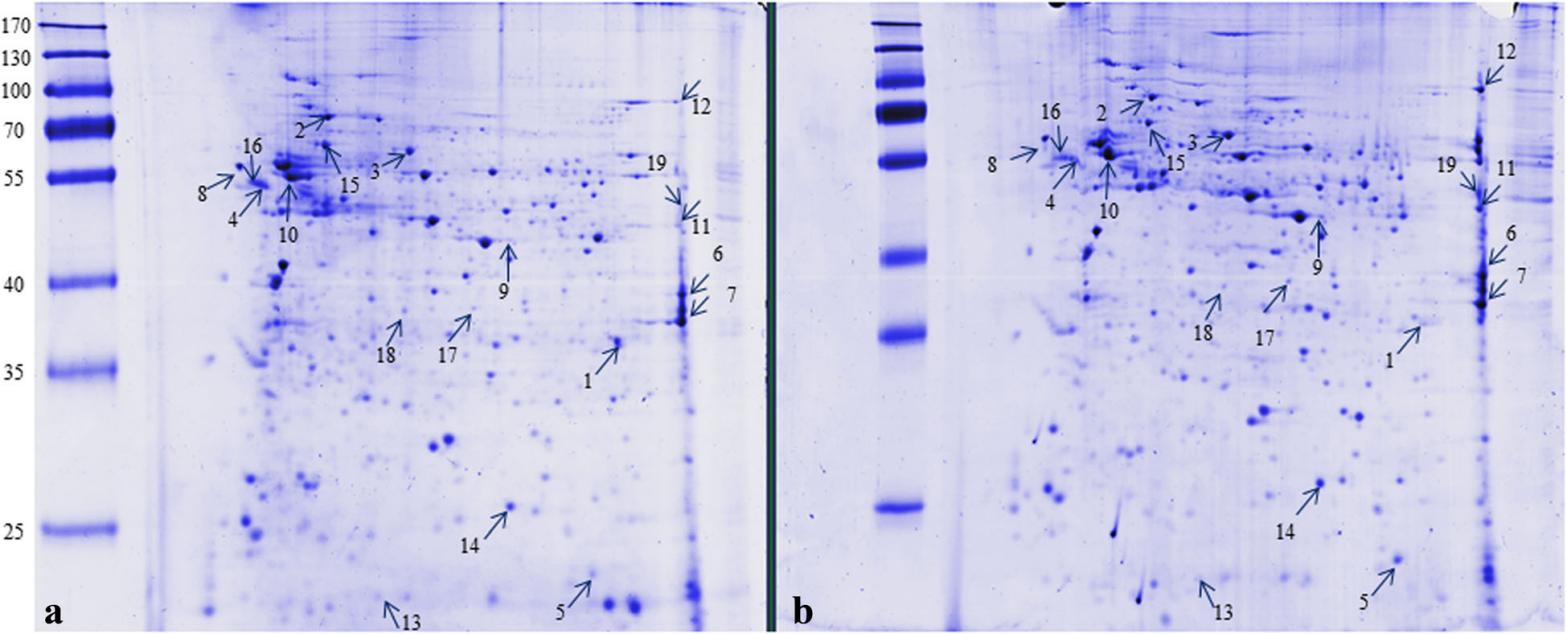
Representative two-dimensional electrophoresis gels of the brain extracts of the female *An. albimanus* mosquitoes: **a** control group; **b** group infected with *Plasmodium berghei* ookinetes. Nineteen differentially expressed proteins were identified (see Table 1 for gel spot number protein identification)

Nineteen differentially expressed proteins were identified with an e-value, score, identity and coverage that are compatible with correct identification and assignment (Fig. 2 and Additional file 1: Table S1). These proteins correspond to 19 genes previously registered in the *An. albimanus* genome data base (https://www.vectorbase.org/taxonomy/anopheles) [23]. The 19 proteins that presented an altered expression level were classified into five categories: ATP synthesis coupled proton transport, cytoskeleton rearrangements and synaptic plasticity, oxidation-reduction process, signal transduction, and miscellaneous (Tables 1 and 2).

**Table 1:** Differentially expressed proteins, post-infection with *P. berghei*

Spot	pI/MW/AA	*An. albimanus*	Protein family	Gene ontology	Fold change
ATP synthesis coupled proton transport					
13	6.1/14.1/127	AALB009730-PA	Vacuolar ATP synthase subunit f	GO:0015991: ATP hydrolysis coupled proton transport	383 Up
19	9.5/107.7/551	AALB005889-PA	F-type H+-transporting ATPase	GO:0015986: ATP synthesis coupled proton transport	6.6 Up
10	4.7/53.7/503	AALB010020-PA	ATP synthase beta subunit	GO:0015986: ATP synthesis coupled proton transporter	2.9 Up
7	6.4/76.2/675	AALB000444-PA	Voltage-dependent anion-selective channel	GO:0055085: Transmembrane transport	2.4 Up
Cytoskeleton rearrangements and synaptic plasticity					
4	4.1/35.6/326	AALB008424-PA	Synapse-associated protein.	GO:0048172: Regulation of short-term neuronal synaptic plasticity	3.7 Down
14	7.2/16.9/147	AALB010134-PA	Cofilin	GO:0030042: Actin filament depolymerization	4.3 Up
16	4.9/59.1/519	AALB010554-PA	Tubulin beta chain	GO:0007017: Microtubule-based process	2.4 Up
Oxidation-reduction process					
1	8.6/66.9/620	AALB010381-PA	Mitochondrial enoyl-CoA hydratase	GO:0055114: Oxidation-reduction process	4.3 Down
11	8.3/61.7/561	AALB006338-PA	Alanine aminotransferase	GO:0030170: Pyridoxal phosphate binding	5.1 Up
6	8.6/52.4/483	AALB000829-PA	Enolase	GO:0006096: Glycolytic process	2.5 Up
3	6.5/69.3/627	AALB008565-PA	Malate oxidoreductase (malic enzyme)	GO:0006108: Malate metabolic process; GO:0055114 oxidation-reduction process	2.2 Up
Signal transduction					
2	6.5/149/1,374	AALB008255-PA	(Hypothetical, HSP70)	GO:0007165: Signal transduction.	3.1 Down
15	5.9/119/1092	AALB008255-PA	HSP70	GO:0007165: Signal transduction	2.5 Down
17	6.0/38.1/340	AALB004447-PA	beta3 G protein	GO:0005515: Guanine nucleotide binding protein	4.4 Up
Miscellaneous					
18	5.6/33.2/316	AALB008188-PA	60S acidic ribosomal protein P0	GO:0005622: Intracellular 60s Ribosomal protein LP0	2.6 Down
9	6.2/45.0/408	AALB004623-PA	Arginine/Creatine kinase	GO:0003824: Molecular function	2.6 Up
5	7.3/15.2/138	AALB007271-PA	Peptidyl-prolyl cis-trans isomerase	GO:0006457: Protein folding	2.3 Up

**Table 2:** Proteins detected only in the brain of mosquitoes infected with *P. berghei*

Spot	pI/MW/AA	*An. albimanus*	Protein family	Gene ontology	Spot intensity
8	4.1/46.5/409	AALB003666-PA	Calreticulin	GO:0005509: Calcium ion binding	1.1
12	8.6/198.3/1,771	AALB002066-PA	Mitofilin	GO:0003676: Nucleic acid binding	2.5

#### ATP synthesis coupled proton transport

During the ookinete invasion of the mosquito midgut, the following proteins were upregulated in the mosquito brain: alpha (AALB010020-PA) and beta (AALB005889-PA) proteins (spots 10 and 19, respectively) of the ATP synthase complex, the vacuolar ATP synthase subunit f (AALB009730-PA) and a voltage-dependent anion-selective channel protein (AALB000444-PA) (spots 13 and 7, respectively).

#### Cytoskeleton rearrangements and synaptic plasticity

The synapse-associated protein (AALB008424-PA), which contains a BTF-2 like transcription factor and a Dos2-like domain (BSD), was downregulated in the brains of infected mosquitoes (spot 4). This protein was found specifically in neurons and is an important molecular element of the nervous system [27, 28].

The tubulin β chain protein (AALB000046-PA) (spot 16) is a key element of microtubule assembly in the cell cytoskeleton [29]. Cofilin (AALB010134-PA) (spot 14) is a protein implicated in regulation of neuronal polarity [30]. Both proteins were upregulated.

Calreticulin (AALB003666-PA) (spot 8), a multifunctional protein mainly involved in directing the proper conformation of proteins, controlling the calcium level, and resisting infection [31], was upregulated during the parasite invasion, potentially as a consequence of mitochondrial stress signalling.

#### The oxidation-reduction process

Enoyl-CoA hydratase (AALB010381-PA) was downregulated during infection (spot 1). This mitochondrial enzyme catalyses the second step in the beta-oxidation pathway of fatty acid metabolism [32]. Another four enzymes of this process were upregulated during infection: (i) malate dehydrogenase (AALB008565-PA) (spot 3), an enzyme that participates in the citric acid cycle, catalysing the oxidation reaction of the malate to oxaloacetate; (ii) enolase (AALB000829-PA) (spot 6), a protein involved in carbohydrate metabolism and energy production; (iii) an alanine aminotransferase (AALB006338-PA) (ALAT, EC 2.6.1.2) (spot 12) that catalyses the transfer of an amino group from L-alanine to α-ketoglutarate and is considered as a marker of tissue damage in various organisms; and (iv) mitofilin (AALB002066-PA) (spot 12), an enzyme that has been reported to counteract sudden oxidative stress.

#### Signal transduction

Downregulation was found for the HSP70 proteins (spots 2 and 15) (AALB008255-PA), which are molecular chaperones that minimize unfolded protein aggregation through degradation or removal [33].

The guanine nucleotide binding protein beta 3 (AALB004447-PA) was upregulated during infection (spot 17). This protein has been shown to be part of the protein complexes allowing for the integration of signals between receptor and effector proteins.

#### Miscellaneous

The 60S acidic P0 ribosomal protein that binds to 25S rRNA (AALB008188-PA) (spot 18) was downregulated during infection, potentially reflecting a higher turnover of the proteins involved in the translation processes.

Two of the proteins that were upregulated during early infection were the enzyme arginine kinase (EC:2.7.3.3) (AALB004623-PA) (spot 9), which catalyses the transfer of phosphate from ATP to arginine, and the enzyme peptidyl-prolyl cis-trans isomerase (spot 5), which is related to high protein folding demand (AALB007271-PA).

Three proteins identified in the *An. albimanus* database represent “hybrid proteins” and potentially correspond to more than one protein (Additional file 2: Figure S1a–c). The annotation of the AALB008255-PA transcript reports a potential protein that presents domains of heat-shock protein 70 in its COOH terminal and arrestin homology in its amino terminal. Since the deduced protein showed a molecular weight of 149.08 kDa and had 1,374 amino acid residues, and the protein herein identified showed a molecular weight of 71.67 kDa, had 656 amino acid residues and was identical to HSP70 of *An. gambiae,* we propose the reannotation of this transcript (Additional file 2: Figure S1a). The AALB000444-PA transcript translation presents porin domains in the amino (NH) terminal as well as proteasome component domains (PCI) in the carboxyl (COOH) terminal. The newly annotated protein would have a molecular weight of 76.21 kDa and 675 amino acid residues. The voltage-dependent anion-selective channel protein presently identified showed a molecular weight of 30.85 kDa and had 282 amino acid residues, identical to the *An. darlingi* protein (Additional file 2: Figure S1b)*.* AALB010381-PA, with a molecular weight of 66.9 kDa and 620 amino acid residues, had domains of the enoyl-CoA hydratase/isomerase, the HSP10–like chaperonin and alcohol dehydrogenase. The Enoyl CoA hydratase herein identified had a molecular weight of 32.28 kDa and 295 amino acid residues. The sequence is identical to that reported for *An. darlingi* (Additional file 2: Figure S1c).

### String analysis

**Fig. 3:**
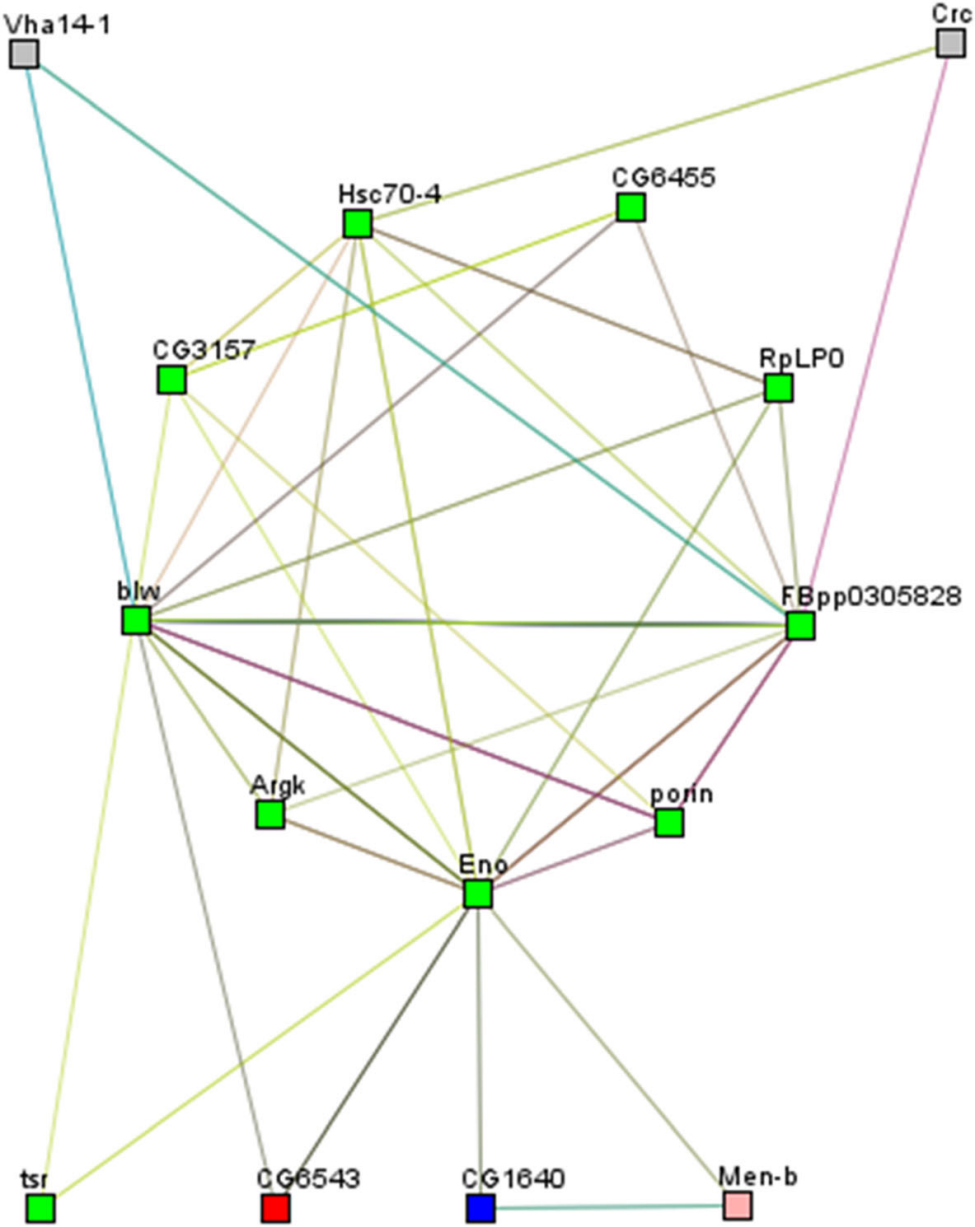
Differentially expressed proteins in the infected *versus* uninfected mosquito brain. Their interaction networks are depicted by STRING and visualized by Medusa. Each node represents the upregulated and downregulated proteins. The edges represent putative protein interactions recorded or predicted by STRING. The graph was obtained using the spectral clustering option, with five clusters. Node rectangles are coloured and the edges predicted functional links. Neighborhood (*green lines*), Gene Fusion (*red lines*), Co-occurrence (*blue lines*), Co-expression (*grey lines*), Experiments (*purple lines*), Databases (*light blue lines*), Textmining (*light green lines*) and Homology (*light grey lines*). *Abbreviations*: CG6543, enolyl-Coa hydratase; CG32683, HSP70; Men-b, malate dehydrogenase; Sap47, synapse-associated protein; CG14715, peptidyl-prolyl cis-trans isomerase; Eno, enolase; Porin, VDAC porin; Crc, calreticulin; Argk, arginine kinase; FBpp0305828, ATP synthase β; CG1640, alanine transaminase; CG6455, mitofilin; Vha14-1, V-type proton ATPase F; Tsr, cofilin; Hsc70-4, HSP70; CG3157, tubulin; GTPase, Gbeta13F protein G β-1; RpLP0, 60s Ribosomal LP0; Blw, F-type H+ transporting ATPase α

Interaction analysis showed that Enolase (Eno) and F-type H+ transporting ATPase α (blw) were molecules with the most interactions (eleven), while ATP synthase β (FBpp0305828) and HSP70 (Hsc70-4) had nine and seven interactions, respectively (Fig. 3).

## Discussion

**Fig. 4:**
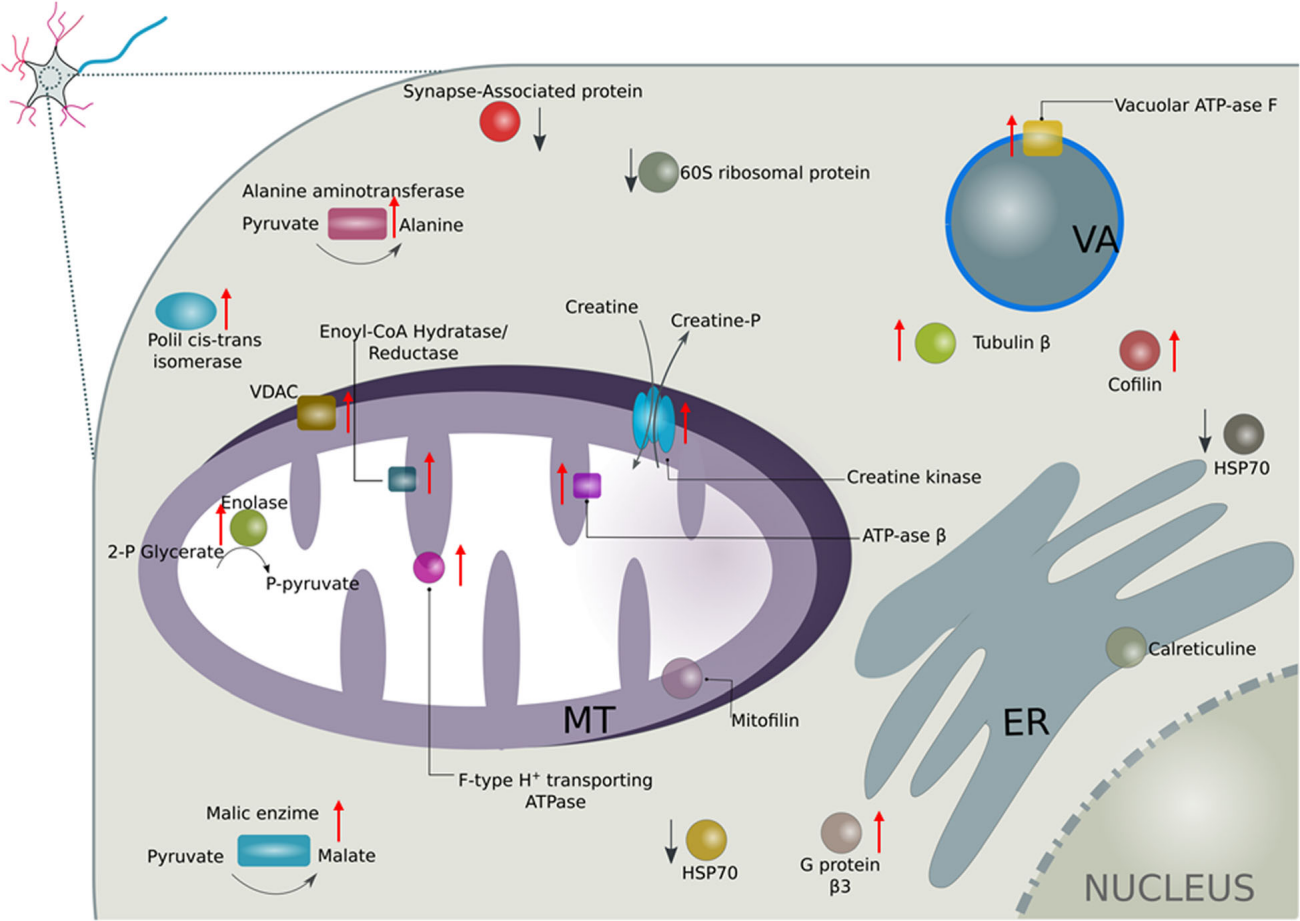
Theoretical intracellular distribution of the 19 differentially expressed proteins. All proteins were located at a synapse. The downregulated proteins are shown with a *downward arrow* (*black*); the upregulated proteins are shown with an *upward arrow* (*red*). *Abbreviations*: MT, mitochondrion; VA, vacuole; ER, endoplasmic reticulum

We herein employed a proteomic approach to analysing the alterations in the protein profile in brains from infected and non-infected mosquitoes. We identified 19 differentially expressed proteins, of which 14 were upregulated and 5 were downregulated. These proteins were mainly associated with the ATP synthesis coupled proton transport, cellular metabolism, cytoskeleton rearrangements, synaptic plasticity and signal transduction. For an overall understanding, the intracellular distribution was defined for the aforementioned 19 proteins (Fig. 4).

In insects, response to stressors are regulated and coordinated by a few hormonal compounds as neuropeptides, ecdysteroids and juvenile hormones. The metabolic response of insects to stress is linked to secretion of biogenic amines in the brain. Within a few minutes after a stress stimulus, reserve energy substrates are mobilized from the fat body. A previous study of *An. gambiae* infected by *P. berghei* showed an alteration in the mosquito head proteome during the progress of infection. Specifically, proteins related to energy metabolism presented differential expression in the brain of sporozoite-infected versus uninfected mosquitoes [15]. Other proteins also showed a differential concentration between infected and uninfected mosquito head extracts, including the synapse-associated protein, the 14-3-3 protein, calmodulin, the stress response protein HSP20 and proteins (e.g. tropomyosin) involved in the cell structure [15]. The proteomic approach presently employed provides a direct portrait of the functional status of the mosquito brain during the early phase of infection by *Plasmodium*. A large number of proteins found to be related to the stress response. The signalling in brain cells (an uninvaded tissue) involved in this response may result from the liberation of intracellular proteins in the haemolymph during the crossing of ookinetes to the basal membrane.

Upregulation of heat-shock proteins (HSPs) during cellular stress, leads to the presence of inducible HSPs such as HSP70 and HSP90 in the haemolymph. These proteins serve as danger signals [34]. Indeed, HSP 70 has been defined as a powerful stress elicitor in many insects. Since these secondary messengers transit through the haemolymph towards all insect tissues, it is expected that brain cells would respond to this danger signal. HSP70 is a family of proteins very strongly upregulated by heat stress and toxic chemicals, particularly heavy metals exposure. However, in anophelines, these proteins also have been associated with dehydration stress [35] and ageing [36]. Surprisingly, during the *Plasmodium* invasion, HSP70 was herein downregulated in the brain of *An. albimanus.*

Several proteins affecting nerve cell function, such as arginine kinase, which were upregulated during *Plasmodium* infection. This protein is involved in energy production and might modulate immune processes *via* changes in the concentration of nitric oxide (NO) and the activity of iNOS, as reported in the scallop *Chlamys farreri* [37]. NO is a well-known immune stress mediator, involved in systemic immune response signalling [13]. Furthermore, NO is liberated during immunological reactions and it is also a neuromodulator coordinating many neuronal activities in insects [38]. Interestingly, phosphoarginine, the end product of the arginine kinase activity, alters the Na (+) - Ca2 (+) exchanger balance [39]. This signalling has been linked to stress signal transduction, affecting the neural function of the squid Na (+) - Ca2 (+) exchange [40]. Other proteomic results have shown that arginine kinase is differentially expressed in the brain of infected versus uninfected of *Gammarus insensibilis* and *G. pulex* [41].

Besides being a structural protein, mitofilin is also involved in protein transport into the mitochondrion and promotes protein import via the mitochondrial intermembrane space assembly pathway [42]. Since the mitochondria may be modified in the neurons of infected mosquitoes, mitofilin may be critical for maintaining the mitochondrial morphology.

The presence of tubulin β chain and cofilin might reflect the reorganization of neurons during infection. Cofilin, is related to the growth of neurons and depolymerization of actin filaments, specifically in neurons and dendritic spines [43]. This protein points to an early neuronal response to *Plasmodium* infection since it has been linked to synapse remodelling [44]. Therefore, cofilin could potentially be a protein responsible or associate for the *Plasmodium-*induced alteration in mosquito behaviour observed in the later stages of the infection.

In *Anopheles gambiae*, the vacuolar ATP synthase subunit f and a voltage-dependent anion-selective channel protein interact with *Plasmodium* enolase mimetic peptide SM-1 [45], a porin whose function in brain cells is still unclear. In our model, *Plasmodium* enolase could has induced upregulation of these proteins. Also, could arise from its interaction with prohibitin, a protein involved in the stress response [46].

The identification of the alpha and beta subunits of the mitochondrial ATP synthase complex might reflect a surge in energy requirements, potentially resulting from the progression of the *Plasmodium* infection.

The protein identified that has a BSD domain related to synapse-associated proteins was previously found in the head of *An. gambiae* infected with *P. berghei* sporozoites [15]. During synaptogenesis of the mouse brain, orthologous synapsis-associated protein 102 (SAP102) is implicated in the transport of ionotropic glutamate receptors to the cell membrane [27]. In *D. melanogaster,* this protein is required for proper behavioural and synaptic plasticity [28]. In anopheline mosquitoes, it may be associated with the same processes described in those organisms.

Calreticulin upregulation could potentially be a consequence of mitochondrial stress signalling during infection. Nevertheless, this protein is also involved in the olfactory system [47], anaesthetic sensitivity [48], and phagocytosis of apoptotic cells [49]. In the innate immune system, calreticulin regulates phagocytosis [50] and encapsulation [51]. In *An. albimanus*, the interaction of this protein with parasite surface molecules has been reported [52].

Enoyl-CoA hydratase also known as crotonase, is essential for metabolizing fatty acids to produce both acetyl CoA and energy. The enoyl-CoA hydratase presently found suggests that the mosquito brain reduces the use of fatty acids as substrates for oxidative energy metabolism during the *Plasmodium* infection.

The malate dehydrogenase reflects a higher energetic demand. Interestingly, this enzyme has been studied as a potential target for species-specific insecticidal compounds [53].

The presence of two spots attributed to the enolase protein, showing different isoelectric points (8.5 and 6.5, respectively) and masses (47.7 0 and 50 kDa), implies that this protein exists in two distinct isoforms.

Alanine aminotransferase (ALAT, EC 2.6.1.2), which participates in maintaining the alanine-proline cycle between flight muscles and fat body during the flight of *Aedes aegypti* [54], is upregulated during injury and toxic substance exposure [55]. Many insects yield lactate and alanine as anaerobic end products, but other species have been known to produce a wide array of other products during hypoxia, including sorbitol, succinate, glycerol, α-glycerol-3-phosphate, pyruvic acid and fatty acids [56]. In *An. stephensi,* infection with *P. yoelii* diminishes the expression of transcripts of two different guanine nucleotide-binding protein genes at 4 hours post-feeding [57]. Hence, it is not surprising that this enzyme is present in the brain cells of *An. albimanus* infected with *P. berghei*. The absence of this protein decreases cellular proliferation and increases apoptosis, due to abnormal mitochondrial function [58].

In the army worm *Spodoptera exigua*, it has been shown that small G proteins regulate part of hemocyte phagocytosis, nodulation and encapsulation through cytoskeletal alteration [59]. In the case of *Anopheles*, guanine nucleotide-binding protein beta 3 (belonging to the small G-protein family) might be related to immune dampening processes.

The diminution of the ribosomal 60S acidic protein would hinder protein synthesis in the mosquito brain. This decrease could limit the amount of energy consumption of the mosquito in order to withstand the energy impounding imposed by *Plasmodium* infection.

Peptidyl-prolyl cis-trans isomerase has been related to high protein folding demand and is upregulated in the midgut of *An. gambiae* (Giles) during the invasion of the o'nyong-nyong virus. Therefore, this protein might constitute a general infection-related responsiveness marker [60] in anophelines during infection.

The STRING program was used to analyse the interactions between the set of 19 brain proteins whose levels were altered during the early stage of a *Plasmodium* infection. Given the scant data available in the anopheline database, we used the orthologous protein from *Drosophila melanogaster*, having defined UniProtKB accession numbers. When the *Drosophila* orthologues of the nineteen regulated proteins were mapped, there was a significant interaction between them. The enolase was determined to be the alfa protein of this interactome. This indicates that the metabolism of neuronal cells is the first cellular process altered by *Plasmodium* infection (Fig. 3). It is noteworthy that the *Drosophila* ortholog proteins identified show up to 60 % identity with the proteins identified in *An. albimanus* (Additional file 3: Table S2). Therefore, it is reasonable to consider that the functionality and interaction between them may be conserved. On the other hand, it is important to perform studies to correlating the proteins expression with behavioural changes in mosquitoes. Several aspects must be considered in further experiments: (i) perform experiments in several stages of the parasite life cycle into mosquitoes; (ii) use other *Plasmodium*-*Anopheles* models; and (iii) analyse the effect of the microbiota in the parasite interaction and the changes in the brain proteome. Finally, our results provide support for future studies using *Plasmodium vivax*, a natural parasite for *Anopheles albimanus*.

## Conclusions

The present results indicate that ookinetes promoted the modification of protein expression in the mosquito brain at 24 hours post-infection. The pattern of differential protein expression in infected versus uninfected mosquitoes indicates that the invasion of midgut tissue by *Plasmodium* triggered a brain response. Whether this change is due to a stress or immune response remains unclear. Nevertheless, we hereby demonstrate that as soon as ookinetes penetrated the intestine, the protein composition of the mosquito brain was altered, indicating potential functional changes. These findings provide novel information about the mechanisms underlying the behaviour and immune response of *Anopheles* during a *Plasmodium* invasion, but there must be continuing study to explore the functional analysis and specific involvement of each of these proteins.

## Additional files

Additional file 1: Table S1. Identification of differentially displayed proteins in *Anopheles albimanus*. (XLSX 23 kb)
Additional file 2: Figure S1. Alignments of the predicted sequence AALB008255-PA from VectorBase data bank, with peptides derived from mass spectra identification (Additional file 1: Table S1). Peptides align only with the carboxyl terminal motif. Box in red indicates initial methionine (ORF) from A. gambiae heat-shock 70 kDa protein AGAP002076-PA. It is highly probable that AALB008255-PA is mischaracterized. (---) indicates no amino acid residues identified and (asterisks) identical amino acid residues. **Figure S2.** Alignments of the predicted sequence AALB000444-PA from VectorBase data bank, with peptides derived from mass spectra identification. Peptides align only with the amine terminal motif and are numbered according to Additional file 1: Table S1. The red box indicates the initial methionine (ORF) of *A. darlingi* COP9 signalosome subunit, ADAC010105-PA. We propose that AALB000444-PA is mischaracterized and corresponds to a voltage-dependent anion-selective channel protein. (---) indicates no amino acid residues identified and (asterisks) identical amino acid residues. **Figure S3.** Alignments of the predicted sequence AALB010381-PA from VectorBase data bank, with peptides derived from mass spectra identification. Peptides are numbered according to Additional file 1: Table S1. Peptides align only with the amine terminal motif and correspond to mitochondrial trans-2-enoyl-CoA hydratase. The red box indicates the initial methionine (ORF) of *Aedes aegypti* zinc binding dehydrogenase protein, AAEL003995-PA. We propose that AALB000444-PA is mischaracterized and corresponds to trans-2-enoyl-CoA reductase. (---) indicates no amino acid residues identified and (asterisks) identical amino acid residues. (DOCX 27 kb)
Additional file 3: Table S2. Identification of differentially displayed proteins in *Anopheles albimanus* and orthologs in *Anopheles darlingi* and *Drosophila melanogaster*. (XLSX 18 kb)

## Abbreviations

2D: Second dimension separation gel
blw: F-typeH+ transporting ATPaseα
BSD: Dos2-like domain
COOH: Carboxyl terminal
Eno: Enolase
GFP: Green fluorescent protein
H_2_O_2_: Hydrogen peroxide
HSP: Heat-shock protein
MS: Mass spectrometry
NH: Amino terminal
NO: Nitric oxide
PCI: Proteasome component domains
pI: Isoelectric point
STRING: Search Toll for the Retrieval of Interacting Genes

## References

[1] WHO (2013). World Malaria Report 2013.

[2] Le BV, Williams M, Logarajah S, Baxter RH (2012). Molecular basis for genetic resistance of *Anopheles gambiae* to *Plasmodium*: structural analysis of TEP1 susceptible and resistant alleles. PLoS Pathog.

[3] Hauck ES, Antonova-Koch Y, Drexler A, Pietri J, Pakpour N, Liu D (2013). Overexpression of phosphatase and tensin homolog improves fitness and decreases *Plasmodium falciparum* development in *Anopheles stephensi*. Microbes Infect.

[4] Garver LS, Bahia AC, Das S, Souza-Neto AJ, Shiao J, Dong Y, Dimopoulos G (2012). *Anopheles* Imd pathway factors and effectors in infection intensity-dependent anti-*Plasmodium* action. PLoS Pathog.

[5] Ramphul UN, Garver LS, Molina-Cruz A, Canepa GE, Barillas-Mury C. *Plasmodium falciparum* evades mosquito immunity by disrupting JNK-mediated apoptosis of invaded midgut cells. Proc Natl Acad Sci USA. 2015;112(5):1273–80. 10.1073/pnas.1423586112 PMC4321252 25552553

[6] Anderson RA, Koella JC, Hurd H (1999). The effect of *Plasmodium yoelii nigeriensis* infection on the feeding persistence of *Anopheles stephensi* Liston throughout the sporogonic cycle. Proc Biol Sci.

[7] Wekesa JW, Copeland RS, Mwangi RW (1992). Effect of *Plasmodium falciparum* on blood feeding behavior of naturally infected *Anopheles* mosquitoes in western Kenya. Am J Trop Med Hyg.

[8] Koella JC, Sorensen FL, Anderson RA (1998). The malaria parasite, *Plasmodium falciparum*, increases the frequency of multiple feeding of its mosquito vector, *Anopheles gambiae*. Proc Biol Sci.

[9] Smallegange RC, van Gemert GJ, van de Vegte-Bolmer M, Gezan S, Takken W, Sauerwein RW, Logan JG (2013). Malaria infected mosquitoes express enhanced attraction to human odor. PLoS One.

[10] Rossignol PA, Ribeiro JM, Spielman A (1984). Increased intradermal probing time in sporozoite-infected mosquitoes. Am J Trop Med Hyg.

[11] Dwivedi SB, Muthusamy B, Kumar P, Kim MS, Nirujogi RS, Getnet D (2014). Brain proteomics of *Anopheles gambiae*. OMICS.

[12] Bendena WG (2010). Neuropeptide physiology in insects. Adv Exp Med Biol.

[13] Herrera-Ortiz A, Martínez-Barnetche J, Smit N, Rodríguez MH, Lanz-Mendoza H (2011). The effect of nitric oxide and hydrogen peroxide in the activation of the systemic immune response of *Anopheles albimanus* infected with *Plasmodium berghei*. Dev Comp Immunol.

[14] Serrano-Pinto V, Acosta-Pérez M, Luviano-Bazán D, Hurtado-Sil G, Batista CV, Martínez-Barnetche J, Lanz-Mendoza H (2010). Differential expression of proteins in the midgut of *Anopheles albimanus* infected with *Plasmodium berghei*. Insect Biochem Mol Biol.

[15] Lefevre T, Thomas F, Schwartz A, Levashina E, Blandin S, Brizard JP (2007). Malaria *Plasmodium* agent induces alteration in the head proteome of their *Anopheles* mosquito host. Proteomics.

[16] Chan AS, Rodriguez MH, Torres JA, Rodriguez Mdel C, Villarreal C (1994). Susceptibility of three laboratory strains of *Anopheles albimanus* (Diptera: Culicidae) to coindigenous *Plasmodium vivax* in southern Mexico. J Med Entomol.

[17] Contreras-Garduño J, Rodríguez MC, Hernández-Martínez S, Martínez-Barnetche J, Alvarado-Delgado A, Izquierdo J (2015). *Plasmodium berghei* induced priming in *Anopheles albimanus* independently of bacterial co-infection. Dev Comp Immunol.

[18] Franke-Fayard B, Trueman H, Ramesar J, Mendoza J, van der Keur M, van der Linden R (2004). A *Plasmodium berghei* reference line that constitutively expresses GFP at a high level throughout the complete life cycle. Mol Biochem Parasitol.

[19] Rodriguez MC, Margos G, Compton H, Ku M, Lanz H, Rodríguez MH, Sinden RE (2002). *Plasmodium berghei*: routine production of pure gametocytes, extracellular gametes, zygotes, and ookinetes. Exp Parasitol.

[20] Shevchenko A, Wilm M, Vorm O, Jensen ON, Podtelejnikov AV, Neubauer G (1996). A strategy for identifying gel-separated proteins in sequence databases by MS alone. Biochem Soc Trans.

[21] Encarnación S, Hernández M, Martínez-Batallar G, Contreras S, Vargas Mdel C, Mora J (2005). Comparative proteomics using 2-D gel electrophoresis and mass spectrometry as tools to dissect stimulons and regulons in bacteria with sequenced or partially sequenced genomes. Biol Proced Online.

[22] A comprehensive, integrated, non-redundant, well-annotated set of reference sequences including genomic, transcript, and protein. Maryland USA. 2016. https://www.ncbi.nlm.nih.gov/refseq/about/nonredundantproteins/#reference. Accessed 8 Feb 2016.

[23] Giraldo-Calderón GI, Emrich SJ, MacCallum RM, Maslen G, Dialynas E, Topalis P (2015). VectorBase: an updated bioinformatics resourse for invertebrate vectors and other organisms related with human diseases. Nucleic Acids Res.

[24] Mascot software from Matrix Science for identification, characterization and quantitation of proteins using mass spectrometry data. London UK. 2012. http://www.matrixscience.com. Accessed 8 Feb 2016.

[25] Szklarczyk D, Franceschini A, Wyder S, Forslund K, Heller D, Huerta-Cepas J (2015). STRING v10: protein-protein interaction networks, integrated over the tree of life. Nucleic Acids Res.

[26] Hooper SD, Bork P (2005). Medusa: a simple tool for interaction graph analysis. Bioinformatics.

[27] Lauks J, Klemmer P, Farzana F, Karupothula R, Zalm R, Cooke NE (2012). Synapse associated protein 102 (SAP102) binds the C-terminal part of the scaffolding protein neurobeachin. PLoS One.

[28] Saumweber T, Weyhersmüller A, Hallermann S, Diegelmann S, Michels B, Bucher D (2011). Behavioral and synaptic plasticity are impaired upon lack of the synaptic protein SAP47. J Neurosci.

[29] Janke C (2014). The tubulin code: molecular components, readout mechanisms, and functions. J Cell Biol.

[30] Sarmiere PD, Bamburg JR (2004). Regulation of the neuronal actin cytoskeleton by ADF/cofilin. J Neurobiol.

[31] Michalak M, Groenendyk J, Szabo E, Gold LI, Opas M (2009). Calreticulin, a multi-process calcium-buffering chaperone of the endoplasmic reticulum. Biochem J.

[32] Zhang J, Ibrahim MM, Sun M, Tang J (2015). Enoyl-coenzyme A hydratase in cancer. Clin Chim Acta.

[33] Feder ME, Hofmann GE (1999). Heat-shock proteins, molecular chaperones, and the stress response: evolutionary and ecological physiology. Annu Rev Physiol.

[34] Zitvogel L, Kepp O, Kroemer G (2010). Decoding cell death signals in inflammation and immunity. Cell.

[35] Benoit JB, López-Martínez G, Phillips ZP, Patrick KR, Denlinger DL (2010). Heat shock proteins contribute to mosquito dehydration tolerance. J Insect Physiol.

[36] Sikulu MT, Monkman J, Dave KA, Hastie ML, Dale PE, Kitching RL (2015). Proteomic changes occurring in the malaria mosquitoes *Anopheles gambiae* and *Anopheles stephensi* during aging. J Proteomics.

[37] Shi X, Wang L, Zhou Z, Yang C, Gao Y, Song L (2012). The arginine kinase in Zhikong scallop *Chlamys farreri* is involved in immunomodulation. Dev Comp Immunol.

[38] Bicker G (2001). Nitric oxide: an unconventional messenger in the nervous system of an orthopteroid insect. Arch Insect Biochem Physiol.

[39] DiPolo R, Berberian G, Beauge L (2004). Phosphoarginine regulation of the squid nerve Na+/Ca2+ exchanger: metabolic pathway and exchanger-ligand interactions different from those seen with ATP. J Physiol.

[40] DiPolo R, Beauge L (1995). Phosphoarginine stimulation of Na(+)-Ca2+ exchange in squid axons--a new pathway for metabolic regulation?. J Physiol.

[41] Ponton F, Lefevre T, Lebarbenchon C, Thomas F, Loxdale HD, Marché L (2006). Do distantly related parasites rely on the same proximate factors to alter the behaviour of their hosts?. Proc Biol Sci.

[42] von der Malsburg K, Müller JM, Bohnert M, Oeljeklaus S, Kwiatkowska P, Becker T (2011). Dual role of mitofilin in mitochondrial membrane organization and protein biogenesis. Dev Cell.

[43] Ng J, Luo L (2004). Rho GTPases regulate axon growth through convergent and divergent signaling pathways. Neuron.

[44] Piccioli ZD, Littleton JT (2014). Retrograde BMP signaling modulates rapid activity-dependent synaptic growth via presynaptic LIM kinase regulation of cofilin. J Neurosci.

[45] Vega-Rodríguez J, Ghosh AK, Kanzok SM, Dinglasan RR, Wang S, Bongio NJ, et al. Multiple pathways for *Plasmodium* ookinete invasion of the mosquito midgut. Proc Natl Acad Sci USA. 2014;111(4):E492–500. 10.1073/pnas.1315517111 PMC3910608 24474798

[46] Ye J, Li J, Xia R, Zhou M, Yu L (2015). Prohibitin protects proximal tubule epithelial cells against oxidative injury through mitochondrial pathways. Free Radic Res.

[47] Stoltzfus JR, Horton WJ, Grotewiel MS (2003). Odor-guided behavior in *Drosophila* requires calreticulin. J Comp Physiol A Neuroethol Sens Neural Behav Physiol.

[48] Gamo S, Tomida J, Dodo K, Keyakidani D, Matakatsu H, Yamamoto D, Tanaka Y (2003). Calreticulin mediates anesthetic sensitivity in *Drosophila melanogaster*. Anesthesiology.

[49] Kuraishi T, Manaka J, Kono M, Ishii H, Yamamoto N, Koizumi K (2007). Identification of calreticulin as a marker for phagocytosis of apoptotic cells in *Drosophila*. Exp Cell Res.

[50] Asgari S, Schmidt O (2003). Is cell surface calreticulin involved in phagocytosis by insect hemocytes?. J Insect Physiol.

[51] Zhang G, Schmidt O, Asgari S (2006). A calreticulin-like protein from endoparasitoid venom fluid is involved in host hemocyte inactivation. Dev Comp Immunol.

[52] Rodríguez Mdel C, Martínez-Barnetche J, Alvarado-Delgado A, Batista C, Argotte-Ramos RS, Hernández-Martínez S (2007). The surface protein Pvs25 of *Plasmodium vivax* ookinetes interacts with calreticulin on the midgut apical surface of the malaria vector *Anopheles albimanus*. Mol Biochem Parasitol.

[53] Pon J, Napoli E, Luckhart S, Giulivi C (2011). Mitochondrial NAD+-dependent malic enzyme from *Anopheles stephensi*: a possible novel target for malaria mosquito control. Malar J.

[54] Belloni V, Scaraffia PY (2014). Exposure to L-cycloserine incurs survival costs and behavioral alterations in *Aedes aegypti* females. Parasit Vectors.

[55] Inagaki Y, Matsumoto Y, Kataoka K, Matsuhashi N, Sekimizu K (2012). Evaluation of drug-induced tissue injury by measuring alanine aminotransferase (ALT) activity in silkworm hemolymph. BMC Pharmacol Toxicol.

[56] Hoback WW, Stanley DW (2001). Insects in hypoxia. J Insect Physiol.

[57] Zhang J, Zhang S, Wang Y, Xu W, Zhang J, Jiang H, Huang F (2014). Modulation of *Anopheles stephensi* gene expression by nitroquine, an antimalarial drug against *Plasmodium yoelii* infection in the mosquito. PLoS One.

[58] John GB, Shang Y, Li L, Renken C, Manella CA, Selker JM (2005). The mitochondrial inner membrane protein mitofilin controls cristae morphology. Mol Biol Cell.

[59] Lee S, Shrestha S, Prasad SV, Kim Y (2011). Role of a small G protein Ras in cellular immune response of the beet armyworm, *Spodoptera exigua*. J Insect Physiol.

[60] Rider MA, Zou J, Vanlandingham D, Nuckols JT, Higgs S, Zhang Q (2013). Quantitative proteomic analysis of the *Anopheles gambiae* (Diptera: Culicidae) midgut infected with o'nyong-nyong virus. J Med Entomol.

