# Supplementary material for: Infection with Plasmodium berghei ookinetes alters protein expression in the brain of Anopheles albimanus mosquitoes
Source: Parasit Vectors. 2016 Oct 11;9:542. doi: 10.1186/s13071-016-1830-9 (PMC5057407; doi:10.1186/s13071-016-1830-9)
Supplement: Additional file 2: Figure S1. — Alignments of the predicted sequence AALB008255-PA from VectorBase data bank, with peptides derived from mass spectra identification (Additional file 1: Table S1). Peptides align only with the carboxyl terminal motif. Box in red indicates initial methionine (ORF) from A. gambiae heat-shock 70 kDa protein AGAP002076-PA. It is highly probable that AALB008255-PA is mischaracterized. (---) indicates no amino acid residues identified and (asterisks) identical amino acid residues. Figure S2. Alignments of the predicted sequence AALB000444-PA from VectorBase data bank, with peptides derived from mass spectra identification. Peptides align only with the amine terminal motif and are numbered according to Additional file 1: Table S1. The red box indicates the initial methionine (ORF) of A. darlingi COP9 signalosome subunit, ADAC010105-PA. We propose that AALB000444-PA is mischaracterized and corresponds to a voltage-dependent anion-selective channel protein. (---) indicates no amino acid residues identified and (asterisks) identical amino acid residues. Figure S3. Alignments of the predicted sequence AALB010381-PA from VectorBase data bank, with peptides derived from mass spectra identification. Peptides are numbered according to Additional file 1: Table S1. Peptides align only with the amine terminal motif and correspond to mitochondrial trans-2-enoyl-CoA hydratase. The red box indicates the initial methionine (ORF) of Aedes aegypti zinc binding dehydrogenase protein, AAEL003995-PA. We propose that AALB000444-PA is mischaracterized and corresponds to trans-2-enoyl-CoA reductase. (---) indicates no amino acid residues identified and (asterisks) identical amino acid residues. (DOCX 27 kb) [file 13071_2016_1830_MOESM2_ESM.docx]

**Figure 1a.**

**AALB008255-PA** MLKEKDDHDLEICKAPSPLSEDRKLNDSDLDPVGSQRVYKKTSSNQLLTLYLGSRELVAR

------------------------------------------------------------

KGVIEPLKGVLYIDSKIINESKIYGQLTLTFRYGREDEEVMGLKFCNEAVIALQQFWPQP

------------------------------------------------------------

VSAEADTLTPLQEALLERLGKNAVPFALEIGTLAPPSVQLLPAKRYTGAPIGTSYDVRVY

------------------------------------------------------------

TAETAGEERVQRRSTVRLGIRLIHKICLDSSKAPAALETATVNNSSFSDAGSSGSASLGP

------------------------------------------------------------

SSGAAGPGPKSPPALVPQSSITDSNANSTIPRALRLRLSPKSLKLSSLHRHSSSVDSTNG

------------------------------------------------------------

GIKSYGDHAVIELTETNKGPQVSVDKPFLWADGRVNLKASLNKAAYVHGENVTVTLDIKN

------------------------------------------------------------

DSRKIVRKIRLVAVQHVDVCMFSNGKFKNIVAEVDVSKHIGPGDTLHASYSLLPVRGTTK

------------------------------------------------------------

NWIAVEGALISSNSADPSSSAYNSKLATSAPRGMLSASVSSSEEKNVFAIYVSYYVKVKL

------------------------------------------------------------

ILSSMGGEVSLKLPFVLGNVELSSANIPRPPDTLSGLKKLRESRRKSSAIASNGGSLDFT

------------------------------------------------------------

PCRSPLSKELSVRDSDEPDEDGTDSSVSRVRTLNLHNQSTPTDGNGRAPHRSLTHDVSPS

------------------------------------------------------------

SLEARGEEQATINSRRELFKNSKFNDNLNSTIDIITEDFQTITANISNLIKSDSSKSNLN

------------------------------------------------------------

EAGTCNQEISVEAQIHCPQLDVAMDDSASNQPSPQRSPQRSPQRSPQPRSEQYNILQISM

------------------------------------------------------------

AAAKAPAVGIDLGTTYSCVGVFQHGKVEIIANDQGNRTTPSYVAFTDTERLIGDAAKNQV

----APAVGIDLGTTYSCVGVFQHGKVEIIANDQGNRTTPSYVAFTDTER-------NQV

********************************************** ***

AMNPTNTIFDAKRLIGRKFDDPAIQADMKHWPFDVESIEGKPKIKVEYKGETKSFFPEEV

AMNPTNTIFDAK------------------------------------------------

************

SSMVLTKMKETAEAYLGKTVTNAVITVPAYFNDSQRQATKDAGTISGLNVLRIINEPTAA

------------------**TVTNAVITVPAYFNDSQR**----------------IINEPTAA

****************** ********

AIAYGLDKKTAGERNVLIFDLGGGTFDVSILSIDDGIFEVKSTAGDTHLGGEDFDNRLVN

AIAYGLDKK--------------------------------**STAGDTHLGGEDFDNRLVN**

********* *******************

HFAQEFKRKHKKDLSTNKRALRRLRTACERAKRTLSSSTQASIEIDSLFEGTDFYTSITR

HFAQEFK--------------------------TLSSSTQASIEIDSLFEGTDFYTSITR

******* ***************************

ARFEELNADLFRSTMEPVEKALRDAKMDKASIHDIVLVGGSTRIPKVQKLLQDFFNGKEL

**ARFEELNADLFR**-----------------**ASIHDIVLVGGSTR**------**LLQDFFNGK**--

************ ************** *********

NKSINPDEAVAYGAAVQAAILHGDKSEEVQDLLLLDVTPLSLGIETAGGVMSVLIKRNTT

--**SINPDEAVAYGAAVQAAILHGDK**-----------------------------------

***********************

IPTKQTQTFTTYSDNQPGVLIQVFEGERAMTKDNNLLGKFELSGIPPAPRGVPQIEVTFD

----**QTQTFTTYSDNQPGVLIQVFEGER**----**DNNLLGKFELSGIPPAPR**----------

************************ ******************

IDANGILNVTALEKSTNKENKITITNDKGRLSKEDIERMVNEAEKYRTEDEKQKETISAK

------------------------------------------------------------

NALESYCFNMKATMEDDKLKDKISDSDKTIVLDKCNDTIKWLDANQLADKEEYEHRQKEL

**NALESYCFNMK**-----------------------------**WLDANQLADKEEYEHR**----

*********** ****************

ESVCNPIISKLYQGAGGAPGGMPGFPGGAPGAAGAGGAAGGAGSGSGPTIEEVD

------------------------------------------------------

**Figure 1b.**

**AALB000444-PA** MAPPSYSDLGKQARDVFNKGYHFGLWKLDVKTKTNSGVEFSTSGHSNQDTGKVFGSLETK

1 -------------------GYHFGLWK---------------------------------

2 ---------------------------------TNSGVEFSTSGHSNQDTGKVFGSLETK

******** ***************************

**AALB000444-PA** YKVKEYGLNFSEKWNTDNTLTSEVSVENQLVKGLKVSFDGMFVPHTGSKTGRFKTAYTHD

3 --VKEYGLNFSEK-----------------------------------------------

4 ----EYGLNFSEKWNTDNTLTSEVSVENQLVK----------------------------

5 -------------WNTDNTLTSEVSVENQLVK----------------------------

6 --------------------------------GLKVSFDGMFVPHTGSK-----------

7 -----------------------------------VSFDGMFVPHTGSK-----------

8 ----------------------------------------------------FKTAYTHD

*********************************************** ********

**AALB000444-PA** RVRVDADFNVDLSGPLVNASGVASYQGWLAGYQVAFDSQKSKITANNFAVGYSAGDFVLH

8 R-----------------------------------------------------------

9 ----------------------------------------SKITANNFAVGYSAGDFVLH

10 ------------------------------------------ITANNFAVGYSAGDFVLH

* ********************

**AALB000444-PA** TNVNDGREFGGLIYQRCNDRLETAVQLSWASGSNATKFGLGAKYDLDKDACVRAKVNNQS

9 TNVNDGR-----------------------------------------------------

10 TNVNDGREFGGLIYQR--------------------------------------------

11 ----------------CNDRLETAVQLSWASGSNATK-----------------------

12 -------------------------------------------YDLDKDACVR-------

13 -------------------------------------------------------VNNQS

************************************* ********** *****

**AALB000444-PA** QIGLGYQQKLRDGVTLTLSTLNIEELTMSVSLSALRNRLAVLTNSSGIHKEQADKYRQLL

13 QIGLGYQQK---------------------------------------------------

14 ---------LRDGVTLTLSTLVDGK-----------------------------------

*************************

DQILLNGEELVETLKLFIEAILNEHVSLVISRQILSDVSFQLTKLPDEISKNVSHFTLDK

------------------------------------------------------------

VQPRVISFEEQVASIRQHLAQIYERNQNWKEAANVLGGIPLETGQKPYSLDYKLETYLKI

------------------------------------------------------------

ARLFLEDEDPVQAESFINRASILQADTKDEKLQILYKVCYARVLDYRRKFIEAAQRYNEL

------------------------------------------------------------

SYRTIVDEGERMTALKKALICTVLASAGQQRSRMLATLFKDERCQHLPAYAILENMYLDR

------------------------------------------------------------

IIRRSELQEFEALLQSHQKATTVDGSTILDRAVFEHNLLSASKLYNNITFEELGALLEIA

------------------------------------------------------------

PPKAERIASQMITEGRMNGYIDQIDGVVHFETREILPMWDKQIQSICYQVNGLIEKIAAA

------------------------------------------------------------

EPEWMNKMIEKEMCP

---------------

**Figure 1c.**

**AALB010381-PA** MANIARLFASRVAQLAQQQQQQQTKLLRLYSSAAPKAYEFIKAELTGEKKNVALITLNRP

--------------------------------------------------NVALITLNRP

**********

KALNALCNGLVAEISDALDRYEADDSIGAIVITGSEKAFAAGADIKEMQPNTYAKCINTD

K**ALNALCNGLVAEISDALDRYEADDSIGAIVITGSEK**------------------**CINTD**

************************************* *****

FLANWTRVAKAQKPIIAAVNGYALGGGCELAMMCDIIYAGDKARFGQPEIALGTIPGAGG

**FLANWTR**-------------------------------------**FGQPEIALGTIPGAGG**

******* ****************

TQRTTRSMGKSKAMEMCLTGNMITAEEAERAGLVSKVVPADKLVAEAVKLGEKISTFSPL

**TQR**--------------------------------------------------**ISTFSPL**

*** *******

IVRLCKEAVNTAYETSLNEGLKFERRHFHATFSTKDRLEG**M**TAFVEKLRQMSLVAKVLRY

**IVR**----------------------**RHFHATFSTK**-------------------------

*** **********

GEFGEPAKVLRLQEEPVPEPKDGEVLIRTLGAPINPADINTIQAVGGNECVGEVIAIGGG

------------------------------------------------------------

DAGATSLKVGDRVVPFATGLGTWRSHAIYSAGQLMKAPAGIGVAEAATITVNPCTGYRML

------------------------------------------------------------

KDFVSLKAGDTVIQNGANSACGQAIIQLCRAWNIECVGVVRDRPEFGQLKDYLKGLGAAE

------------------------------------------------------------

ILTEEELRTTKLFRDGIFRKPKLALNCVGGKNALEMSRQLDQAGVMVTYGGMSREPVTVP

------------------------------------------------------------

TASLIFKDLRFVGFWMTRWTKENAASPARAEMFNELFGLIDRGALKAPAHEMIAFDEYIS

------------------------------------------------------------

AVTNALNIQGFVGKKYIFKF

--------------------
